# Metabolomic Signatures and Advanced Echocardiography Highlight Clinical Risk and Early Cardiac Changes in Systemic Lupus Erythematosus: Six-Year Follow-Up

**DOI:** 10.3390/metabo16020131

**Published:** 2026-02-13

**Authors:** Nicola Campana, Michele Migliari, Antonio Deidda, Martino Deidda, Luca Fazzini, Gianmario Usai, Giulia Anna Maria Luigia Costanzo, Antonio Noto, Cristina Piras, Davide Firinu, Stefano Del Giacco, Luigi Atzori, Christian Cadeddu Dessalvi

**Affiliations:** 1 Department of Medical Sciences and Public Health, University of Cagliari, 09042 Cagliari, Italy; nicolacampana93@gmail.com (N.C.); michele.migliari1@gmail.com (M.M.); antonio.deidda93@gmail.com (A.D.); martino.deidda@tiscali.it (M.D.); luca.fazzini10@gmail.com (L.F.); g.usai3@studenti.unica.it (G.U.); giuliaam.costanzo@unica.it (G.A.M.L.C.); davide.firinu@unica.it (D.F.); delgiacco@unica.it (S.D.G.); 2 Department of Biomedical Sciences, University of Cagliari, 09042 Cagliari, Italy; antonionoto@unica.it (A.N.); cristina.piras@unica.it (C.P.); latzori@unica.it (L.A.)

**Keywords:** systemic lupus erythematosus, metabolomics, ^1^H-NMR spectroscopy, GC-MS, right ventricular remodeling, 3D echocardiography, cardiovascular involvement, longitudinal study, metabolic fingerprint, autoimmune disease

## Abstract

**Background/Objectives:** Cardiovascular involvement drives morbidity and mortality in systemic lupus erythematosus (SLE). Echocardiography has limited predictive value for long-term outcomes, and subclinical right ventricular (RV) remodeling is poorly characterized. Metabolic dysregulation may influence immune activation and myocardial injury. This study investigates whether baseline metabolomic profiles are associated with longitudinal RV changes and disease progression in SLE. **Methods:** In this prospective, single-center study, patients with established SLE and no known cardiac disease underwent baseline clinical assessment, plasma metabolomic profiling, and advanced echocardiography, including 3D RV analysis. Echocardiography was repeated after 6 years. Metabolomics was performed using NMR spectroscopy and GC–MS. Disease progression was assessed via the SLICC/ACR damage index (SDI), defining clinical stability as ΔSDI = 0 and worsening as ΔSDI ≥ 1. **Results:** Twenty-five patients completed the follow-up (88% female; mean age 51 ± 13 years). Despite normal echocardiographic values, subtle but significant RV changes were observed, remaining within reference ranges, including mild declines in fractional area change and septal longitudinal strain (*p* < 0.05). Clinically worsened patients showed reduced TAPSE, while stable patients had slight increases (*p* < 0.05). Multivariate metabolomic analysis distinguished stable from worsened patients (R^2^Y = 0.772; Q^2^ = 0.483), primarily driven by higher 2-aminoheptanedioic acid values in those with progression (*p* < 0.05), along with trends toward higher fumarate and lower fructose and glucopyranose. **Conclusions:** Baseline metabolomic and advanced echocardiographic profiling may identify SLE patients at risk of disease progression. Longitudinal echocardiography enables monitoring of subtle RV changes, supporting personalized surveillance to detect early subclinical trajectories before overt dysfunction develops.

## 1. Introduction

Systemic lupus erythematosus (SLE) is a chronic autoimmune disease characterized by persistent inflammation and multisystem involvement, with cardiovascular complications representing a major determinant of long-term morbidity and mortality [1,2,3,4,5]. Beyond overt clinical manifestations, patients with SLE frequently develop subclinical cardiovascular alterations that may precede symptomatic disease by several years [4,6,7]. Chronic immune activation, endothelial dysfunction, metabolic derangements, and long-term exposure to immunomodulatory therapies synergistically contribute to cardiac remodeling and functional impairment, even in the absence of traditional cardiovascular risk factors [1,8,9,10].

While left ventricular (LV) involvement in SLE has been extensively investigated, the RV has received comparatively limited attention [11,12]. This gap is clinically relevant, as RV structure and function are emerging as strong predictors of outcomes across a wide range of cardiovascular conditions [13,14]. In patients with SLE, RV remodeling may be influenced by immune-mediated myocardial injury, pulmonary vascular involvement, and subtle alterations in myocardial energetics [11,12]. Conventional two-dimensional echocardiography is limited in its ability to accurately characterize RV geometry and function, whereas three-dimensional (3D) echocardiography allows more precise and reproducible assessment of RV volumes and systolic performance [5,11,12].

Metabolomics has emerged as a powerful tool for capturing downstream biochemical alterations resulting from the interaction between genetic susceptibility, immune activation, and environmental factors in multiple settings [15,16,17,18,19,20]. In SLE, metabolic reprogramming has been linked to immune cell dysfunction, oxidative stress, and altered energy metabolism, potentially contributing to cardiovascular involvement [17,18,21]. Beyond classical inflammatory pathways, increasing evidence highlights a key role of antiphospholipid antibodies and membrane-microdomain-mediated signaling in immune-driven cardiovascular damage. In particular, lipid rafts have emerged as critical signaling platforms modulating endothelial activation, thrombosis, and inflammatory responses in antiphospholipid syndrome and SLE [22].

Moreover, recent studies have shown that non-criteria antiphospholipid antibodies and atypical antibody profiles, including so-called “seronegative” APS, are clinically relevant and associated with thromboinflammatory manifestations and subclinical cardiovascular risk, further contributing to immune heterogeneity and cumulative organ damage in autoimmune diseases [23].

However, most metabolomic studies on SLE have focused on disease activity or organ-specific damage, with limited integration of advanced cardiac imaging and a paucity of long-term longitudinal data [7,24,25].

To date, no longitudinal studies have systematically integrated 3D echocardiographic assessment of RV remodeling with comprehensive metabolomic profiling in patients with SLE. The absence of such an integrated approach limits mechanistic understanding of subclinical cardiac involvement and hampers the identification of metabolic signatures associated with long-term structural and functional changes in the RV.

Therefore, the aim of the present study is to investigate whether clinical disease worsening over a 6-year prospective follow-up in patients with SLE is associated with baseline advanced metabolomic and echocardiographic profiles, assessed by ^1^H-NMR, gas chromatography–mass spectrometry, and 3D echocardiography, as well as with longitudinal changes in advanced echocardiographic parameters reflecting long-term RV remodeling.

## 2. Materials and Methods

### 2.1. Study Design and Population

This was a prospective, single-center longitudinal study including patients with an established diagnosis of SLE according to the SLICC classification criteria. At baseline (2019), patients underwent clinical evaluation, conventional and 3D transthoracic echocardiography, and blood sampling for metabolomic analysis.

**Figure 1:**
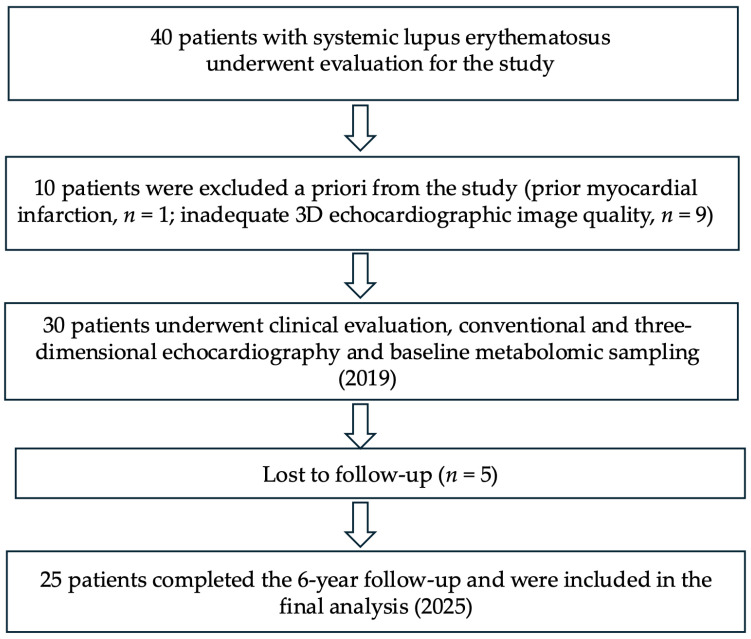
Study flowchart. Of the 40 SLE patients initially enrolled, 10 were excluded a priori (9 due to inadequate echocardiographic image quality and 1 due to prior myocardial infarction), and 5 were lost to follow-up. The study population therefore comprised 25 patients with complete baseline clinical, echocardiographic, and metabolomic data, who also underwent clinical evaluation and 3D echocardiographic reassessment at 6-year follow-up.

A total of 40 patients were initially enrolled. Of these, 9 patients were excluded due to inadequate echocardiographic image quality precluding reliable 3D analysis, 1 patient was excluded because of a history of prior myocardial infarction, and 5 patients did not attend follow-up visits. The final analysis included 25 patients with complete baseline clinical, echocardiographic, and metabolomic data, and 6-year follow-up clinical and 3D echocardiographic reassessment. The study flow and patient selection process are summarized in Figure 1.

Inclusion criteria were age ≥ 18 years, confirmed SLE diagnosis, availability of baseline metabolomic samples, and adequate 3D echocardiographic datasets at baseline and follow-up. Exclusion criteria included known cardiomyopathy, moderate-to-severe valvular heart disease, non-SLE-related pulmonary hypertension, advanced chronic kidney disease, pregnancy, or inadequate acoustic window.

The study was conducted in accordance with the Declaration of Helsinki and approved by the local Institutional Review Board. Written informed consent was obtained from all participants.

### 2.2. Clinical Assessment

At baseline, all patients underwent a comprehensive clinical evaluation including medical history, physical examination, and standard laboratory testing. Demographic and anthropometric variables (age, sex, height, weight, body mass index [BMI]), blood pressure, disease duration, and treatment history were recorded.

Disease-related variables included SLE duration and organ damage assessed by the SLICC/ACR damage index (SDI) [26,27,28]. For outcome classification, patients were considered clinically stable if no increase in the SDI was observed at follow-up (ΔSDI = 0), while clinical progression was defined as an increase of at least one point (ΔSDI ≥ 1) [28]. This threshold was chosen a priori based on established SLE literature. The SLICC/ACR damage index was specifically designed to capture irreversible organ injury, and multiple longitudinal cohorts have demonstrated that any increase in SDI, even from 0 to 1, is associated with higher risk of subsequent damage accrual and increased mortality. For this reason, prevention of any new SDI point is considered a key therapeutic target, and ΔSDI ≥ 1 is the standard operational definition of damage accrual in major SLE inception and follow-up studies [29,30,31].

### 2.3. Echocardiographic Assessment

#### 2.3.1. Conventional Echocardiography

Transthoracic echocardiography was performed using commercially available ultrasound systems equipped with phased-array transducers (Vivid E80, GE Healthcare, Chicago, IL, USA), following ASE/EACVI recommendations [32,33]. Standard two-dimensional (2D) measurements included LV end-diastolic diameter, interventricular septal and posterior wall thickness, LV ejection fraction (LVEF), left atrial volume index (LAVI), transmitral E and A waves, tissue Doppler E′, and E/E′ ratio.

Right heart assessment included RV basal diameter in the apical four-chamber view, right atrial area, tricuspid annular plane systolic excursion (TAPSE), tricuspid lateral S′ velocity, fractional area change (FAC), and estimated pulmonary artery systolic pressure (PAPs).

#### 2.3.2. Three-Dimensional Echocardiography of the Right Ventricle

3D datasets of the RV were acquired from a modified apical four-chamber view using matrix-array transducers (Vivid E80, GE Healthcare), ensuring full inclusion of the RV inflow, apical region, and outflow tract. Single-beat or multi-beat acquisitions were used according to image quality and heart rhythm. Care was taken to avoid stitching artifacts and to optimize visualization of the RV free wall [34,35].

Offline analysis was performed using dedicated software (4D RV-Function 2.0, a module of TomTec-Arena; TomTec Imaging Systems, Unterschleissheim, Germany). Every RV full-volume 3D dataset automatically produced three standard views (planes): four-chamber, coronal, and sagittal. An operator traced the endocardial border at end-diastole and end-systole for the three predefined RV planes, and then an automated border detection algorithm completed the tracking. When needed, the same operator corrected the endocardial borders frame-by-frame to minimize artifacts. Papillary muscles of the tricuspid valve, moderator band and endocardial trabeculae were considered part of the RV cavity. After automatically tracking RV contours over the entire cardiac cycle by 3D technology, we obtained the RV end-diastolic and end-systolic volume (RV EDV and ESV, respectively), RV EF and stroke volume (SV) and RV FAC. Furthermore, we derived both the RV-free lateral wall (RVLS Free) and the interventricular septum (RVLS Septum) 3D STE longitudinal Strain (LS) [36,37,38,39]. Measurements were indexed to body surface area when appropriate.

Although reproducibility was not formally assessed within the present study, advanced echocardiographic measurements were obtained by experienced operators using standardized acquisition and analysis protocols in accordance with current guideline-recommended reference values [33,40].

### 2.4. Metabolomic Analysis

#### 2.4.1. Sample Collection and ^1^H-NMR Spectroscopy

Fasting venous blood was collected at baseline (2019), processed within two hours, and plasma samples were stored at −80 °C until analysis. The extraction of water-soluble metabolites from plasma samples was performed based on the Folch, Lees and Sloane-Stanley procedure and has been already described in previous papers of our group [41]. 400 μL of plasma was dissolved in 1.2 mL of a chloroform/methanol mixture (1:1, *v*/*v*) and 175 mL of H_2_O. The solution was finally centrifuged at 4500× *g* rpm and 4 °C for 30 min, and ~1 mL of hydrophilic phase containing the hydrophilic components was separated from the lipophilic one, dried using a speed vacuum concentrator (Eppendorf, Hamburg, Germany) and then stored at −80 °C. Dried hydrophilic plasma extracts were re-dissolved in 690 μL of potassium phosphate buffer in D_2_O (0.1 M, pH 7.4) and 10 μL of TSP as internal standard (98 atom % D, Sigma-Aldrich, Milan, Italy). An aliquot of 650 μL was analyzed by ^1^H-NMR. ^1^H-NMR experiments were conducted using a Varian UNITY INOVA^®^ 500 spectrometer operating at 499.839 MHz for protons and equipped with a 5 mm double-resonance probe (Agilent Technologies, Santa Clara, CA, USA). ^1^H-NMR spectra were acquired with a spectral width of 6000 Hz, a 90° pulse, an acquisition time of 2 s, a relaxation delay of 2 s, and 256 scans. A presaturation sequence was used to suppress the residual H_2_O signal with low power radiofrequency irradiation for 2 s. ^1^H-NMR spectra were imported into an ACDlab Processor Academic Edition (version 12.01, 2010, Advanced Chemistry Development, Toronto, ON, Canada) and pre-processed with line broadening of 0.1 Hz, zero-filled to 64 K, prior to Fourier transformation. Spectra were manually phased and baseline corrected and chemical shifts referenced internally to TSP at δ = 0.0 ppm. All samples were processed and analyzed using the same standardized protocol, thereby limiting technical variability and potential batch effects.

#### 2.4.2. Gas Chromatography–Mass Spectrometry (GC-MS)

Complementary untargeted metabolomic profiling was performed by GC-MS. Before analysis, plasma samples were thawed at room temperature. To form a pooled sample for quality control and an average composition sample to analyze, we collected 100 μL of each sample. 400 μL of plasma were treated with 1200 μL of cold methanol in 2 mL Eppendorf tubes, vortex mixed and centrifuged for 10 min at 14,000× *g* rpm (16.9 G)*. 400 μL of the upper phase were transferred in glass vials (1.5 mL) and evaporated to dryness overnight in an Eppendorf vacuum centrifuge. 50 μL of a 0.24 M (20 mg/mL) solution of methoxylamine hydrochloride in pyridine was added to each vial; samples were vortex mixed and left to react for 17 h at room temperature in the dark. Then, 50 μL of MSTFA (N-Methyl-N-trimethylsilyltrifluoroacetamide) were added and left to react for 1 h at room temperature. As internal standard, the derivatized samples were diluted with hexane (100 μL) with tetracosane (0.01 mg/mL), just before GC-MS analysis.

Instrumental parameters: Samples were analyzed using an Agilent 5975C (Agilent Technologies, Santa Clara, CA, USA) interfaced to the GC 7820 (new 5977B/7890B) equipped with a DB-5ms column (J & W), injector temperature at 230 °C, detector temperature at 280 °C, helium carrier gas flow rate of 1 mL/min. The GC oven temperature program was 90 °C initial temperature with 1 min hold time and ramping at 10 °C/min to a final temperature of 270 °C with 7 min hold time. One μL of the derivatized sample was injected in split (1:4) mode. After a solvent delay of 3 min, mass spectra were acquired in full scan mode using 2.28 scans/s with a mass range of 50–700 Amu [42].

Mass spectral deconvolution: Each acquired chromatogram was analyzed using the free software AMDIS, version 2.68 (Automated Mass Spectral Deconvolution and Identification System; http://chemdata.nist.gov/mass-spc/amdis, accessed on 25 November 2025) that identified each peak by comparison of the relative mass spectra and the retention times with those stored in an in-house-made library comprising 255 metabolites. Other metabolites were identified using NIST08 (National Institute of Standards and Technology’s mass spectral database) and the Golm Metabolome Database (GMD, (http://gmd.mpimp-golm.mpg.de, accessed on 25 November 2025)). Through this approach, 113 compounds were accurately identified, while 28 other metabolites were tentatively assigned, relying on GMD and NIST libraries. AMDIS analysis produced an Excel datasheet that was successively subjected to chemometric analysis.

### 2.5. Statistical Analysis

Continuous variables were expressed as mean ± standard deviation (SD) when normally distributed, or median and interquartile range (IQR) otherwise. Categorical variables were presented as counts and percentages. Normality was assessed using the Shapiro–Wilk test. Student’s *t*-test was used for continuous variables with a Gaussian distribution, whereas the Mann–Whitney U test was applied to continuous variables with a non-Gaussian distribution; Fisher’s exact test was used for binary variables. A two-sided *p*-value < 0.05 was considered statistically significant.

Specific analyses were performed to exclude the influence of cardiovascular risk factors and ongoing therapies on SLE worsening (ΔSDI over time).

Echocardiographic changes were analyzed and interpreted as continuous variables. No predefined echocardiographic cut-offs were used to define “functional worsening”, as all parameters were primarily evaluated in terms of longitudinal change over time rather than categorical abnormality. Reference ranges were used only to contextualize the clinical meaning of observed changes.

Prior to multivariate analysis, metabolomic data were preprocessed to reduce technical variability and improve model robustness. Metabolite peak areas were log-transformed to reduce heteroscedasticity and Pareto-scaled to balance the contribution of low- and high-abundance metabolites. Missing values, when present, were imputed using a k-nearest neighbors approach.

For orthogonal partial least squares discriminant analysis (OPLS-DA) modeling, patients were classified according to clinical disease progression at follow-up, defined as an increase in the SDI (ΔSDI ≥ 1). This outcome was used as the dependent variable (Y) in the multivariate analysis. Model performance was evaluated by explained variance (R^2^X, R^2^Y) and predictive ability (Q^2^), estimated through cross-validation.

Model robustness and the absence of overfitting were further evaluated by permutation testing (500 times). The ion plot then displays the correlation coefficient between the original y-variable and the permuted y-variable on the *x*-axis versus the cumulative R^2^ and Q^2^ on the *y*-axis and draws the regression line. The intercept is a measure of the overfit, Q^2^Y intercept value less than 0.05 is indicative of a valid model.

Metabolites with a variables important in the projection (VIP) value higher than >1 were selected for evaluation of their role in class separation. The metabolites characterized by VIP > 1, potentially responsible for the separation between the two classes, were evaluated with a Mann–Whitney U test, with *p*-values adjusted for multiple comparisons using the Benjamini–Hochberg false discovery rate (FDR) procedure.

## 3. Results

### 3.1. Study Population

Of the 40 SLE patients initially enrolled, 10 were excluded a priori (9 due to inadequate echocardiographic image quality and 1 due to prior myocardial infarction). Of the remaining 30 patients, 5 were lost to follow-up. The final analysis included 25 patients with complete baseline clinical, echocardiographic, and metabolomic data, and 6-year follow-up clinical and 3D echocardiographic reassessment.

The mean age was 51 ± 13 years, with predominantly females (88%); mean BMI was 23 ± 3.3 kg/m^2^, mean disease duration was approximately 27 [10–30] years, and systolic and diastolic blood pressures were within the normal range. Detailed clinical and anthropometric characteristics are reported in Table 1.

Baseline exposure to corticosteroids, hydroxychloroquine, and immunosuppressive therapies, as well as cardiometabolic risk factors and renal involvement, were comparable between patients with and without SDI progression. No significant differences were observed for smoking status, diabetes, hypertension, lipid profile, eGFR < 60 mL/min/1.73 m^2^, or medication exposure (Table 2).

### 3.2. Changes in Conventional Echocardiographic Parameters

Baseline conventional echocardiography showed normal LV size and preserved systolic function. Over 6 years, LV diameters and LVEF remained stable, with no significant changes in LV end-diastolic diameter or LVEF (66 [60–68] % vs. 64 [61–66] %, *p* = 0.49). However, subtle structural changes were observed. Interventricular septal thickness increased from 8 mm [7.7–9.6] to 9 mm [8–10] (*p* = 0.04), while LV posterior wall thickness remained unchanged. Right heart conventional echocardiographic parameters showed a significant increase in RV basal diameter in the apical four-chamber view (30 [29–32] mm to 33 [31–34] mm, *p* = 0.03). Right atrial area showed a non-significant trend toward enlargement. TAPSE and tricuspid S′ velocity exhibited modest, non-significant changes over time (TAPSE: 20 [19–23] to 19 [18–21] mm, *p* = 0.25; S′: 13 [12–15] to 12 [11–13] cm/s, *p* = 0.07), while estimated PAPs remained stable.

Changes in conventional echocardiographic parameters between baseline and 6-year follow-up are summarized in Table 3.

### 3.3. Three-Dimensional Right Ventricular Remodeling

3D echocardiography demonstrated modest but statistically significant longitudinal changes in RV volumes over follow-up. Baseline RVEDV was 55.4 [43.3–59.8] mL, increasing to 61 [54–74] mL at 6 years (*p* = 0.01, +11%). RVESV increased from 23.9 [20.2–26.2] mL to 28.4 [23.3–32.2] mL (*p* = 0.012, +18%). Importantly, despite these volumetric changes, median RV volumes remained within accepted reference ranges [43]. RVEF remained globally preserved and within the normal range (baseline 56.6 [54.9–58] % vs. 55.2 [51–59] % at follow-up, *p* = 0.6). In contrast, deformation suggested early functional changes, with a gradual directional reduction over time. Specifically, septal right ventricular longitudinal strain decreased from −22.8 [−20.9–−25] % to −19.5% [−17.8–−23.2] (*p* = 0.02, +14.5%), while free-wall strain showed a non-significant trend toward reduction (−29.1 [−26.1–−32.5] % to −27.4 [−24.3–−31.7] %, *p* = 0.37). FAC declined modestly from 51.9 ± 5.3% to 48.9 ± 6.7% (*p* = 0.05), with mean values remaining above commonly accepted abnormality thresholds.

Overall, these findings indicate progressive but mild RV remodeling with preserved global systolic function and subtle changes in longitudinal mechanics, largely within reference limits, consistent with early or subclinical structural adaptation rather than overt RV dysfunction.

Three-dimensional RV volumetric and functional parameters at baseline and 6-year follow-up are reported in Table 4.

### 3.4. Relationship Between RV Function and Clinical Damage

Clinical disease progression, defined as an increase in SDI score ≥ 1, was associated with a reduction in TAPSE (−3.3 ± 4.3 mm), whereas stable patients showed a modest increase (+1 ± 2.8 mm; *p* = 0.007). Patients with an SDI increase over 6 years experienced a mild but significant decline in RV systolic function compared with stable patients. This decline was not evident in the overall cohort, suggesting that subtle RV dysfunction may be masked unless cumulative organ damage is considered.

### 3.5. Metabolomic Profiling and Discriminant Metabolites

Baseline metabolomic profiling identified 113 plasma metabolites by GC-MS and additional 28 putatively annotated compounds, complemented by ^1^H-NMR-derived metabolites. Multivariate analysis using OPLS-DA modeling revealed a correlation between baseline metabolomic profile and disease progression, showing significant clustering between clinically worsened and stable patients; however, it did not show significant clustering with right ventricular functional remodeling.

**Figure 2:**
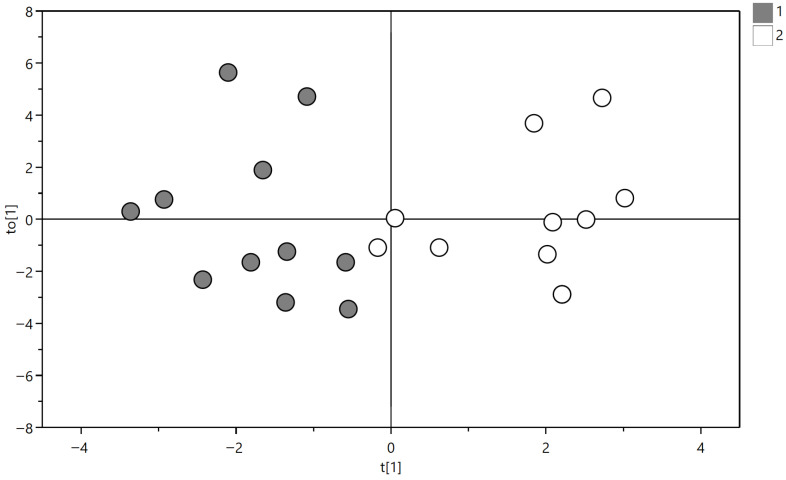
OPLS-DA score plot based on baseline metabolomic profiles. Group 1 (gray circles) includes patients with stable disease, whereas Group 2 (white circles) includes patients with progression of cumulative organ damage (increase in SDI Damage Index). The model (1 predictive and 1 orthogonal component) includes patients with progression of cumulative organ damage (increase in SLICC Damage Index) and shows a clear separation between patients with stable disease and those with clinical and functional progression over 6 years (R^2^X = 0.386, R^2^Y = 0.772, Q^2^ = 0.483).

Multivariate analysis using OPLS-DA with baseline metabolomic data demonstrated robust separation between patients with subsequent clinical worsening (increase in SLICC score; group 1) and those with stable disease (group 2), as shown in Figure 2.

**Figure 3:**
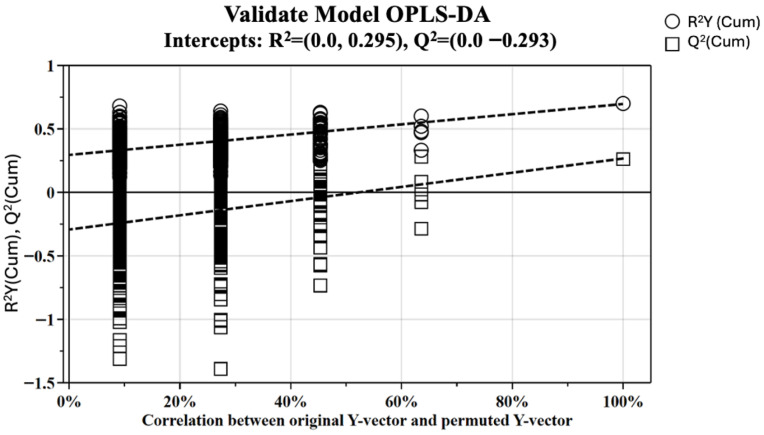
Validation plots of OPLS-DA model using the permutation test. The horizontal axis shows the correlation between the permuted and actual data, while the vertical axis displays the cumulative values of R^2^ and Q^2^. The intercept gives an estimate of the overfitting phenomenon. The Q^2^ intercept value of −0.293 indicates the statistical validity of the OPLS-DA model.

The OPLS-DA model was established with one predictive and two orthogonal components and shows good values of R^2^X, R^2^Y, and Q^2^ (0.386, 0.772 and 0.483). The validity of the OPLS-DA model was evaluated through a permutation test (Figure 3) using 500 permutations. The Q2 intercept value of −0.293 indicates the statistical validity of the OPLS-DA model (Table 5).

**Figure 4:**
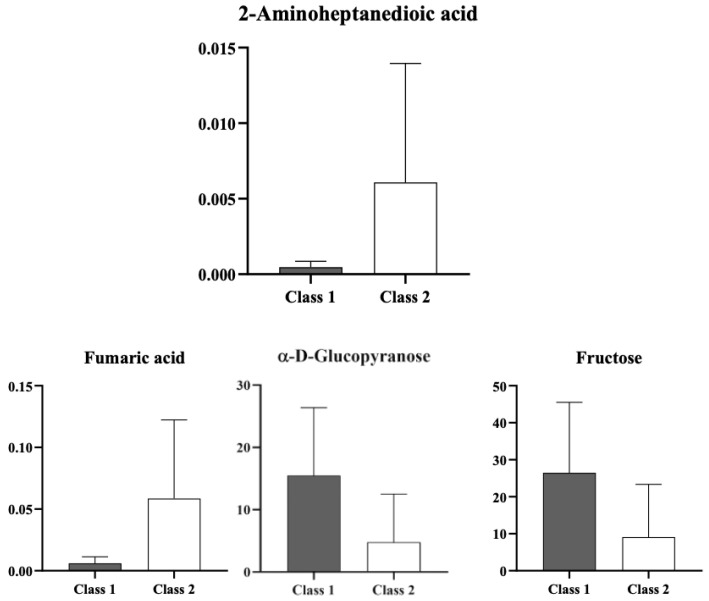
Gray columns represent patients with stable disease (Group 1), while white columns represent patients with progression of cumulative organ damage (ΔSDI ≥ 1). Patients in the progression group (white columns) exhibited significantly higher levels of 2-aminoheptanedioic acid (*p* < 0.05) and a trend toward increased fumaric acid (*p* = 0.08), whereas patients with stable disease (gray columns) showed a tendency toward higher levels of fructose (*p* = 0.08) and glucopyranose (*p* = 0.08).

Among the most discriminant metabolites, significative higher levels of 2-aminoheptanedioic acid (*p* < 0.05) and a trend toward increased fumaric acid (*p* = 0.08) and decreased fructose (*p* = 0.08) and glucopyranose (*p* = 0.08) characterized patients with clinical disease progression. The main discriminant metabolites contributing to group separation are reported in Figure 4.

## 4. Discussion

To our knowledge, this is the first study to provide a long-term clinical and advanced echocardiographic assessment in combination with metabolomic profiling in SLE, in which we prospectively examined the relationship between baseline metabolomic signatures, advanced echocardiographic parameters, and disease progression over a 6-year follow-up.

### 4.1. Right Ventricular Remodeling as a Subclinical Phenotype in SLE

Although overt cardiac manifestations in SLE are well recognized, subclinical myocardial involvement remains underdiagnosed. Prior studies have predominantly focused on LV or pericardial abnormalities, with limited attention to the RV [4,44,45,46]. Our findings indicate that even in the absence of clinical cardiovascular disease, patients with long-standing SLE develop progressive RV enlargement and subtle deterioration of deformation indices, while maintaining preserved ejection fraction and values that remain within normal limits. The pattern dilation with impaired strain has been described in early stages of cardiomyopathy and pulmonary vascular disease, suggesting that RV functional reserve may be compromised before conventional markers become abnormal [47,48]. Notably, these changes occurred with most parameters remaining within accepted reference ranges, indicating mild structural and mechanical adaptation rather than established RV dysfunction. Although these alterations are subtle and do not directly correlate with clinical progression, their observation over time underscores the importance of serial echocardiographic monitoring to detect early subclinical remodeling that may precede future organ involvement in patients with SLE.

### 4.2. Association Between Organ Damage Progression and RV Dysfunction

Our findings indicate that subtle changes in RV function occur in parallel with clinical disease progression in SLE patients. This was observed as increases in the SLICC/SDI score were accompanied by a pronounced decline in TAPSE, whereas TAPSE showed a slight increase in patients with stable disease. The use of ΔSDI ≥ 1 as the definition of disease worsening reflects the clinically validated concept of damage accrual in SLE, where even a single new damage item has been consistently associated with worse long-term prognosis and increased mortality in longitudinal cohorts. These observations support the notion that cumulative systemic injury is closely linked to myocardial involvement, consistent with the hypothesis that chronic inflammation, immune dysregulation, microvascular alterations, and long-term corticosteroid exposure may contribute to subtle cardiomyopathic processes in SLE, highlighting the importance of serial echocardiographic monitoring to capture early functional changes before overt cardiac manifestations become clinically apparent [49].

### 4.3. Integration of Baseline Metabolomics and Echocardiography with Clinical Progression over Time

Our results demonstrate that combining baseline advanced echocardiographic assessment with metabolomic profiling effectively identifies the subset of SLE patients most likely to experience disease progression. Metabolomic analysis revealed a significant correlation between baseline echocardiographic parameters, metabolites, and disease worsening, with clear clustering of patients who clinically worsened versus those who remained stable (R^2^Y = 0.772; Q^2^ = 0.483). Variable importance in projection (VIP) analysis indicates that this separation was driven by distinct metabolic fingerprint with higher 2-aminoheptanedioic acid levels (*p* < 0.05) and trends toward increased fumaric acid and decreased fructose and glucopyranose (all *p* = 0.08) in patients with clinical disease progression. These findings emphasize the value of integrating baseline metabolic and advanced echocardiographic data for characterizing patterns of disease progression in SLE. Furthermore, they underscore the broader applicability of multi-omics approaches, combining metabolic and imaging biomarkers, not only in SLE but also in other systemic conditions, to provide a comprehensive understanding of disease pathophysiology and progression.

The biochemical interpretation of the data derived from OPLS-A and VIP analyses is complex and, while it should be approached with caution, provides interesting insights. Statistical results offer useful prognostic tools and, despite the limitations related to the small sample size, the limited availability of previous data, and the absence of metabolomic evaluations at follow-up, it is possible to formulate some pathophysiological hypotheses based on biochemical knowledge and existing literature.

2-aminoheptanedioic acid, also known as α-aminopimelic acid or α-aminopimelate, is an α-amino acid. Although there is currently no direct evidence linking its levels to immunological mechanisms in SLE, it is known that alterations in amino acid metabolism in T cells influence disease pathophysiology by regulating proliferation, differentiation, and cellular function through pathways such as mTORC1, glutaminolysis, and nucleotide biosynthesis [50,51]. The changes observed in 2-aminoheptanedioic acid levels in our data therefore suggest a potential link with immunometabolic processes in SLE, providing a rationale for future mechanistic studies.

Similarly, alterations in metabolites such as fructose, glucopyranose, and fumarate —although not statistically significant but showing interesting trends that might emerge in larger cohorts—suggest that clinical disease worsening may be associated with modifications in carbohydrate metabolism and tricarboxylic acid (TCA) cycle intermediates, consistent with immunometabolic and cardiometabolic mechanisms implicated in SLE. In chronic autoimmune activation, immune and vascular cells undergo metabolic reprogramming that couples inflammatory signaling to glycolytic flux, mitochondrial function, and redox balance, while long-term exposure to glucocorticoids and other therapies can further modulate systemic glucose homeostasis [18,52]. In parallel, the elevation of fumarate is biologically plausible beyond its role as a TCA intermediate: fumarate can accumulate under mitochondrial stress and can covalently modify cysteine residues in proteins (succination), thereby linking mitochondrial state to redox-sensitive signaling and inflammatory programs [53,54]. In the myocardium, sustained inflammatory stress is also associated with changes in substrate utilization and energetic efficiency, with a relative shift toward carbohydrate use being described in cardiometabolic and heart failure settings; these concepts provide a mechanistic bridge between the observed carbohydrate/TCA signals and the subtle RV remodeling captured by longitudinal echocardiography [55,56]. Taken together, the findings are best framed at the pathway level as evidence for differential regulation of glycolysis/TCA coupling and carbohydrate handling between groups, consistent with immunometabolic and cardiometabolic mechanisms implicated in SLE-associated organ damage and cardiovascular remodeling [1,2,18].

In this context, emerging data further support a mechanistic link between immune heterogeneity, thromboinflammatory pathways, and subclinical cardiovascular involvement in SLE. Non-criteria antiphospholipid antibody profiles, increasingly recognized in “seronegative” APS, may contribute to endothelial dysfunction and microvascular injury even in the absence of overt thrombosis, potentially interacting with metabolic and inflammatory pathways captured by our metabolomic profiling [22,23].

### 4.4. Clinical Implications

The findings of our study suggest that the integration of baseline metabolomic profiling and advanced echocardiographic assessment serves two complementary functions in the evaluation of patients with SLE. First, at baseline, this combined approach allows for risk stratification, identifying patients with metabolic and echocardiographic profiles indicative of higher intrinsic susceptibility to disease progression, even before overt clinical deterioration occurs. Second, during a longitudinal follow-up, serial echocardiographic monitoring enables the detection of two types of changes: on one hand, it reveals subclinical alterations—such as modest increases in RV volumes, borderline reductions in FAC, and subtle impairments in septal strain—that are not yet pathological in absolute terms but may represent early myocardial injury not yet reflected in clinical outcomes; on the other hand, it captures mild systolic functional changes, particularly reductions in TAPSE, which are associated with clinical worsening over time. Taken together, these two functions—baseline stratification and dynamic longitudinal assessment—provide a framework for understanding disease progression and for guiding tailored monitoring strategies in SLE patients.

### 4.5. Limitations

This study has several limitations.

The sample size is limited, reflecting the monocentric design and the long-term nature of a follow-up. Selection bias cannot be entirely excluded, as patients with inadequate image quality or missing follow-up data were excluded from the analysis.

A further limitation of the study is the lack of formal blinding procedures and quantitative assessment of inter- and intra-observer reproducibility for advanced echocardiographic measurements, although image analyses were performed by multiple experienced operators following standardized protocols.

In addition, SDI scores at baseline and follow-up were recorded only as cumulative totals, preventing a detailed analysis of damage in specific organ systems.

Although all eligible SLE patients from our reference outpatient clinics in southern Sardinia were consecutively enrolled, the absence of a formal power calculation limits the statistical robustness and generalizability of our findings, as the sample reflects only the accessible population in this specific region.

Additionally, the study did not integrate markers of endothelial dysfunction, microvascular disease, or cardiopulmonary interaction, which may also contribute to RV remodeling.

An important limitation of the present study is that metabolomic profiling was performed only at baseline. The absence of repeated metabolomic assessments during the follow-up precludes the evaluation of temporal trajectories of metabolic changes and does not allow reconstruction of individual metabolic curves over time. Therefore, we cannot determine whether the observed metabolic signatures are stable traits, progressive alterations, or secondary epiphenomena of disease evolution or treatment exposure. Accordingly, the present design does not allow any inference on causal relationships between specific metabolic pathways and subsequent RV remodeling or disease progression. Our findings should therefore be interpreted as identifying prognostic metabolic patterns rather than etiopathogenetic mechanisms. Longitudinal metabolomic studies with repeated sampling will be required to clarify whether changes in these pathways precede, accompany, or result from cardiac remodeling and cumulative organ damage.

### 4.6. Future Perspectives

The findings of the present study open several avenues for future research.

First, the relatively small, single-center cohort highlights the need for validation in larger, multicenter populations, in order to confirm the robustness and generalizability of the observed associations between metabolomic profiles and clinical progression in SLE, and between longitudinal changes in echocardiographic parameters and clinical disease progression.

Second, although baseline metabolomic profiling provided valuable prognostic insights, longitudinal metabolomic assessment would be essential to determine whether temporal changes in metabolic signatures parallel or precede the progression of RV structural and functional alterations. Repeated metabolomic sampling over time in a large patient population may enable a pathways analysis and the identification of dynamic metabolic trajectories associated with disease activity, cumulative organ damage, and cardiac involvement.

Third, future studies should aim to integrate metabolomics with other biomarkers of cardiovascular risk, including circulating inflammatory mediators, endothelial dysfunction markers, and cardiac-specific biomarkers, as well as advanced imaging techniques such as cardiac magnetic resonance. A truly multimodal approach may enable a more comprehensive characterization of subclinical myocardial involvement in SLE.

Interventional studies exploring metabolic modulation, lifestyle interventions, or pharmacological strategies aimed at improving myocardial energetic efficiency could help clarify whether modifying these pathways translates into improved cardiac outcomes in SLE.

## 5. Conclusions

Baseline metabolomic profiling, combined with prospective echocardiographic monitoring, represents a promising tool for the follow-up of SLE patients. Our study shows that this integrated approach can identify patients at higher risk of disease progression and detect early subclinical trajectories of RV remodeling before overt dysfunction develops. Serial echocardiography allows for the tracking of functional changes over time, including early systolic impairments associated with clinical worsening. Overall, these findings support the potential of combining metabolic and imaging biomarkers to guide risk-adapted monitoring and improve understanding of disease trajectories in SLE.

## Figures and Tables

**Table 1:** Baseline Clinical and Anthropometric Characteristics of the Study Population (*n* = 25). Continuous variables are expressed as mean ± standard deviation (SD) for normally distributed data or as median [interquartile range, IQR] for non-normally distributed data. Normality of distributions was assessed using the Shapiro–Wilk test.

VARIABLE	VALUE
AGE (YEARS)	51 ± 13
SEX (FEMALE/MALE)	22/3
BODY MASS INDEX (KG/M2)	23 ± 3.3
SYSTOLIC BLOOD PRESSURE (MMHG)	115 [105–120]
DIASTOLIC BLOOD PRESSURE (MMHG)	71 ± 10
DISEASE DURATION (YEARS)	27 [10–30]

**Table 2:** Baseline cardiovascular risk factors, medication exposure, and key clinical confounders in the study population. The table summarizes baseline cardiovascular risk factors, renal involvement, and medication exposure, and additional analyses were performed to exclude their influence on SLE clinical worsening (ΔSDI over time). No significant differences were observed between patients with and without SDI progression, indicating a balanced distribution of major clinical confounders at baseline. Continuous variables are expressed as mean ± standard deviation or median [interquartile range], according to data distribution. Categorical variables are presented as counts (percentages). Comparisons between groups were performed using Student’s *t*-test or Mann–Whitney U test for continuous variables and Fisher’s exact test for categorical variables, as appropriate.

	BASELINE— TOTAL POPULATION	BASELINE— ΔSDI ≥ 1 AT FU	BASELINE— ΔSDI = 0 AT FU	*p* VALUE
SMOKING STATUS	(7/25) 20%	(3/13) 25%	(4/12) 33%	0.9
TYPE 2 DIABETES MELLITUS	(1/25) 4%	(1/13) 8%	(0/12) 0%	1
BODY MASS INDEX (KG/M2)	23 ± 3.8	22.5 ± 2.9	24 ± 4.5	0.31
HYPERTENSION	(3/25) 12%	(2/13) 15%	(1/12) 8%	1
e-GFR < 60 ML/MIN	(0/25) 0%	(0/13) 0%	(0/12) 0%	1
HYPERCHOLESTEROLEMIA	(5/25) 20%	(2/13) 17%	(3/12) 25%	1
NON–HDL CHOLESTEROL (MG/DL)	121 ± 31 mg/dL	112 ± 38 mg/dL	128 ± 23 mg/dL	0.25
CORTICOSTEROID THERAPY	(21/25) 84%	(10/13) 77%	(11/12) 92%	1
PREDNISONE DOSAGE	7.5 [5–12.5] mg	8.75 [5–12.5] mg	6.75 [5–12.5] mg	0.57
HYDROXYCHLOROQUINE THERAPY	(11/25) 44%	(6/13) 46%	(5/12) 42%	1
HYDROXYCHLOROQUINE DOSAGE	200 [200–400] mg	300 [200–400] mg	200 [200–250]	0.92
AZATHIOPRINE THERAPY	(7/25) 28%	(2/13) 15%	5/12 (12) 42%	0.4
AZATHIOPRINE DOSAGE	150 [125–187.5] mg	50 [50–50] mg	150 [90–150] mg	0.36
METHOTREXATE THERAPY	(4/25) 16%	(2/13) 15%	(2/12) 17%	1
METHOTREXATE DOSAGE	10 [10–12.5] mg	10 [10–10] mg	15 [15–15] mg	0.82
MYCOPHENOLATE MOFETIL THERAPY	(1/25) 4%	1 (1/13) 8%	(0/12) 0%	1
BELIMUMAB THERAPY	(1/25) 4%	1 (1/13) 8%	(0/12) 0%	1
BASELINE SDI	2.5 [1–4]	0 [0–1]	0.5 [0–2.25]	0.38

Legend: e-GFR = estimated Glomerular Filtration Rate; FU = follow-up; SDI = Systemic Lupus International Collaborating Clinics Damage Index. All values refer to baseline measurements. Baseline ΔSDI ≥ 1 at FU indicates baseline values in patients who experienced disease worsening at follow-up (ΔSDI ≥ 1), whereas Baseline ΔSDI = 0 at FU indicates baseline values in patients who remained clinically stable at follow-up (ΔSDI = 0).

**Table 3:** Changes in conventional echocardiographic parameters between baseline (2019) and follow-up (2025). Echocardiographic measurements obtained at baseline and at 6-year follow-up in the overall study population (n = 25). Continuous variables are reported as mean ± SD or median [IQR], according to data distribution. Normality was assessed using the Shapiro–Wilk test. Paired comparisons between baseline and follow-up measurements were performed using the paired Student’s *t*-test for normally distributed variables and Mann–Whitney U test for non-normally distributed variables.

PARAMETER	BASELINE	FOLLOW-UP	*p*-VALUE
LVEDD (MM)	45 ± 3.9	44.7 ± 4.4	0.59
INTERVENTRICULAR SEPTUM (MM)	8 [7.7–9.6]	9 [8–10]	0.04
POSTERIOR WALL THICKNESS (MM)	8 [7–9]	8 [8–9.3]	0.65
LVEF (%)	66 [60–68]	64 [61–66]	0.49
LAVI (ML/M2)	27.1 [22.3–31.7]	26.1 [22.4–31.5]	0.75
E/A RATIO	1.09 [0.88–1.2]	0.9 [0.8–1.2]	0.33
E/E′ RATIO	7.85 [7–9.23]	6.7 [5.4–8.3]	0.03
RV BASAL DIAMETER, 4C (MM)	30 [29–32]	33 [31–34]	0.03
RA AREA (CM2)	12.5 [11–14.6]	13.3 [11–15]	0.65
TAPSE (MM)	20 [19–22.5]	19 [18–21]	0.3
S′ VELOCITY (CM/S)	13 [12–15]	12 [11–13]	0.07
ESTIMATED PAPS (MMHG)	22.5 [19.8–26]	23 [20–27]	0.52

Legend: LVEDD, left ventricular end-diastolic diameter; LVEF, left ventricular ejection fraction; LAVI, left atrial volume indexed to body surface area; RV, right ventricle; RA, right atrium; PAPS, systolic pulmonary arterial pressure.

**Table 4:** Three-dimensional right ventricular volumetric and functional parameters at baseline and follow-up. Right ventricular structural and functional parameters assessed at baseline and at 6-year follow-up in the study population (n = 25). Continuous variables are expressed as mean ± standard deviation (SD) for normally distributed data or as median [interquartile range, IQR] for non-normally distributed data. Normality of distributions was assessed using the Shapiro–Wilk test. Paired comparisons between baseline and follow-up measurements were performed using the paired Student’s *t*-test for normally distributed variables and the Mann–Whitney U test for non-normally distributed variables. A two-sided *p* value < 0.05 was considered statistically significant.

PARAMETER	BASELINE	FOLLOW-UP	*p*-VALUE
RVEDV (ML)	55.4 [43.3–59.8]	61.4 [54.1–74.1]	0.01
RVESV (ML)	23.9 [20.2–26.2]	28.4 [23.3–32.2]	0.01
RVEF (%)	56.6 [54.9–58]	55.2 [51–59]	0.6
RVLS SEPTAL (%)	−22.8 [−20.9–−25]	−19.5 [−17.8–−23.2]	0.02
RV FREE-WALL STRAIN (%)	29.1 [26.1–32.5]	27.4 [24.3–31.7]	0.37
FAC (%)	51.9 ± 5.3	48.9 ± 6.7	0.05

Legend: RV, right ventricle; RVEDV, right ventricular end-diastolic volume; RVESV, right ventricular end-systolic volume; RVEF, right ventricular ejection fraction; RVLS, right ventricular longitudinal strain; FAC, fractional area change.

**Table 5:** Metabolites identified in plasma samples with VIP > 1. Metabolites with VIP > 1, potentially driving the separation between the two groups, were compared using the Mann–Whitney U test, and the resulting *p*-values were corrected for multiple testing using the Benjamini–Hochberg false discovery rate (FDR) method.

METABOLITES	VIP VALUE	VIPCV VALUE *	*p*-VALUE	*p*-VALUE CORRECT **
2-AMINOHEPTANEDIOIC ACID	1.58	1.26	0.0006	0.0072
GLUCOSE	1.41	1.42	0.0473	0.1135
FUMARIC ACID	1.33	1.06	0.0192	0.0768
A-D-GLUCOPYRANOSE	1.29	1.46	0.0281	0.0843
FRUCTOSE	1.24	1.60	0.0190	0.0768
MYRISTIC ACID	1.21	1.08	0.1330	0.2280
SORBOSE	1.18	1.25	0.0759	0.1518
SERINE	1.17	0.95	0.3476	0.3792
PROLINE	1.17	0.91	0.3316	0.3792
ERYTHRONIC ACID	1.10	1.20	0.1713	0.2570
THREONINE	1.04	0.94	0.2509	0.3345
GLYCINE	1.04	0.92	0.9487	0.9487

Legend: * VIPcv: VIP computed from the selected cross-validation round; ** *p*-values were subjected to the Benjamini–Hochberg adjustment.

## Data Availability

The raw data supporting the findings of this study are available from the corresponding author upon reasonable request, as further analyses of the data are currently underway for future studies.

## Abbreviations

The following abbreviations are used in this manuscript:

SLE	Systemic Lupus Erythematosus
RV	Right Ventricle/Right Ventricular
LV	Left Ventricle/Left Ventricular
3D	Three-Dimensional
2D	Two-Dimensional
RVEDV	Right Ventricular End-Diastolic Volume
RVESV	Right Ventricular End-Systolic Volume
RVEF	Right Ventricular Ejection Fraction
FAC	Fractional Area Change
RVLS	Right Ventricular Longitudinal Strain
TAPSE	Tricuspid Annular Plane Systolic Excursion
S′	Tricuspid Annular Systolic Velocity
PAPs	Pulmonary Artery Systolic Pressure
LVEF	Left Ventricular Ejection Fraction
LVEDD	Left Ventricular End-Diastolic Diameter
LAVI	Left Atrial Volume Index
BMI	Body Mass Index
SLEDAI-2K	Systemic Lupus Erythematosus Disease Activity Index 2000
SLICC	Systemic Lupus International Collaborating Clinics
SDI	SLICC/ACR Damage Index
GC–MS	Gas Chromatography–Mass Spectrometry
^1^H-NMR	Proton Nuclear Magnetic Resonance
OPLS-DA	Orthogonal Partial Least Squares Discriminant Analysis
VIP	Variable Importance in Projection
TCA	Tricarboxylic Acid (cycle)
FDR	False Discovery Rate
ASE	American Society of Echocardiography
EACVI	European Association of Cardiovascular Imaging
